# Tailoring Chitosan/LTA Zeolite Hybrid Aerogels for Anionic and Cationic Dye Adsorption

**DOI:** 10.3390/ijms22115535

**Published:** 2021-05-24

**Authors:** Martina Salzano de Luna, Francesco Greco, Raffaele Pastore, Giuseppe Mensitieri, Giovanni Filippone, Paolo Aprea, Domenico Mallamace, Francesco Mallamace, Sow-Hsin Chen

**Affiliations:** 1 Department of Chemical, Materials and Production Engineering, University of Naples Federico II, Piazzale Tecchio 80, 80125 Napoli, Italy; francesco.greco@unina.it (F.G.); raffaele.pastore@unina.it (R.P.); mensitie@unina.it (G.M.); gfilippo@unina.it (G.F.); paolo.aprea@unina.it (P.A.); 2 Departments of ChiBioFarAm and MIFT-Section of Industrial Chemistry, University of Messina, CASPE-INSTM, V.le F. Stagno d’Alcontres 31, 98166 Messina, Italy; mallamaced@unime.it; 3 Department of Nuclear Science and Engineering, Massachusetts Institute of Technology, Cambridge, MA 02139, USA; francesco.mallamace@unime.it (F.M.); sowhsin@mit.edu (S.-H.C.)

**Keywords:** adsorption, chitosan, zeolite, molecular interactions, water purification

## Abstract

Chitosan (CS) is largely employed in environmental applications as an adsorbent of anionic dyes, due to the presence in its chemical structure of amine groups that, if protonated, act as adsorbing sites for negatively charged molecules. Efficient adsorption of both cationic and anionic dyes is thus not achievable with a pristine chitosan adsorbent, but it requires the combination of two or more components. Here, we show that simultaneous adsorption of cationic and anionic dyes can be obtained by embedding Linde Type A (LTA) zeolite particles in a crosslinked CS-based aerogel. In order to optimize dye removal ability of the hybrid aerogel, we target the crosslinker concentration so that crosslinking is mainly activated during the thermal treatment after the fast freezing of the CS/LTA mixture. The adsorption of isotherms is obtained for different CS/LTA weight ratios and for different types of anionic and cationic dyes. Irrespective of the formulation, the Langmuir model was found to accurately describe the adsorption isotherms. The optimal tradeoff in the adsorption behavior was obtained with the CS/LTA aerogel (1:1 weight ratio), for which the maximum uptake of indigo carmine (anionic dye) and rhodamine 6G (cationic dye) is 103 and 43 mg g^−1^, respectively. The behavior observed for the adsorption capacity and energy cannot be rationalized as a pure superposition of the two components, but suggests that reciprocal steric effects, chemical heterogeneity, and molecular interactions between CS and LTA zeolite particles play an important role.

## 1. Introduction

Synthetic dyes are widely used in various sectors, such as the textile, leather, and paper industry, and it has been estimated that thousands of tons of different dyes are produced yearly [1]. If not properly treated, the resulting wastewaters may cause serious damages to human health and the environment. Due to the large volumes of dyed wastewaters, the recalcitrant nature of most dyes and their high tendency to contaminate water even in small concentrations, water pollution by dyes is considered a serious environmental issue [2,3,4,5]. According to a recent review study by Yaseen and Scholz, dye concentration in wastewater may significantly vary, depending on the location and the adopted purification line, and it ranges from tens to thousands of mg L^−1^ [6].

Among the possible removal strategies, adsorption is claimed as one of the most feasible, versatile, and low-cost methods, relying on the physical and/or chemical interactions between the targeted molecules and the adsorbing substrate [7]. The main characteristics of an ideal adsorbent material are large specific surface area, high porosity, great adsorption capacity, as well as simplicity of production, stability, and low cost and environmental impact [8].

In the last years, a great deal of research has been devoted to the identification of novel materials that can be effectively exploited for water remediation purposes [9,10,11]. Biopolymers emerged as environmentally friendly candidates for adsorbent development, and chitosan (CS), a polysaccharide obtained by deacetylation of chitin, particularly stepped out as one of the most promising materials [12]. Chitosan, indeed, has been already successfully exploited as a dye adsorbent in a number of different forms, such as beads [13,14], films [15], gels [16,17], and aerogels [18,19,20]. Among them, aerogels are often preferred because of their highly porous structure that can absorb large amounts of water, thus allows to maximize the interactions of chitosan with the pollutant molecules [21].

The chemical structure of chitosan is responsible for its effectiveness as an adsorbent. It is indeed characterized by the presence of amine groups that acquire a net positive charge when in protonated form, and thus work as active sites for adsorption of negatively charged molecules, mainly thanks to electrostatic interactions and, to a lesser extent, to hydrogen-bonding interactions [12]. Although extremely effective in the adsorption of anionic pollutants, chitosan is not able to bind and remove cationic dyes. Due to the great variety of synthetic dyes present in wastewater, however, the adsorbents have to be effective towards as many species as possible, in order to ensure a high detoxification level even when multiple pollutants are present [22]. In this sense, alternative strategies need to be proposed. Because of the inherent difficulty in effectively combining different functionalities in a single hybrid/composite adsorbent material, indeed, the literature on broad-spectrum adsorbents is still relatively underdeveloped. Actually, only a few examples exist of adsorbent materials that are effective in removing both anionic and cationic species in the same testing conditions [16,19,22,23].

The present work fits in this frame, as it is focused on the preparation of broad-spectrum adsorbents that are able to effectively remove both anionic and cationic dyes. To this aim, hybrid chitosan-based aerogels were developed by adding Linde Type A (LTA) zeolite particles. Zeolites are hydrated aluminosilicates with a regular and open crystalline structure that can accommodate water molecules and different positively charged ions/molecules, making them suitable for ion-exchange and adsorption processes [24]. Although their original and main use in the field of water treatment is related to heavy metals removal, several studies recently also investigated their dye adsorption ability. In the present study, chitosan and zeolite particles are combined into hybrid adsorbents (CS/LTA) obtained by ice-templating.

## 2. Materials and Methods

### 2.1. Aereogel Preparation

**Figure 1:**
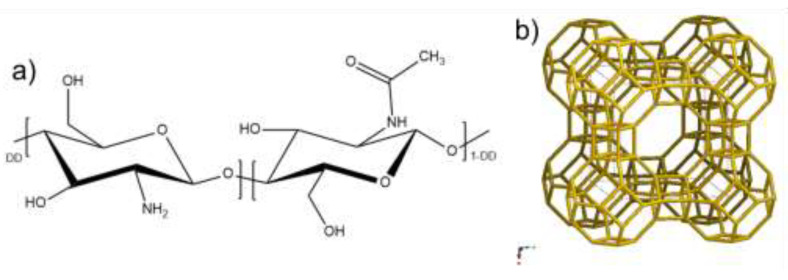
(**a**) Chemical formula of chitosan highlighting the glucosamine (left) and N-acetyl glucosamine (right) units (DD = deacetylation degree) and (**b**) crystal lattice of LTA zeolite (taken from the database of the International Zeolite Association).

CS powder (78% ca. of deacetylation degree and 200–300 kDa, by Sigma Aldrich) was added to a 98/2 *v*/*v*% solution of bi-distilled water and acetic acid and kept under stirring overnight. Then, the pH was adjusted to ~5 and the CS solution was mixed with a suspension of zeolite LTA (by Carlo Erba Reagenti) with formula of Na_2_O·Al_2_O_3_·2SiO_2_·4.5H_2_O. The CS formula and LTA crystal lattice are reported in Figure 1.

The total amount of chitosan and zeolite in the aqueous mixture was fixed to 20 mg mL^−1^, but different CS/LTA weight ratios were investigated, namely 1:2, 1:1, and 2:1. The CS/LTA mixture was kept under mechanical stirring for 30 min to get a homogenous dispersion. After the addition of an aqueous solution of glutaraldehyde, the CS/LTA dispersion was immediately poured into a polydimethylsiloxane mold and frozen at −25 °C overnight in a refrigerator. The samples were then lyophilized in a vacuum freeze-dryer for three days. Finally, the obtained aerogels were subject to a thermal treatment (90 °C, 1 h) to allow the crosslinking reaction in CS [25].

### 2.2. Aereogel Characterization

Rheological experiments were performed with a stress-controlled rotational rheometer (ARG2, TA Instruments). Time sweep experiments (25 °C, 1 rad s^−1^) in cone-plate geometry (diameter 40 mm, angle 2°) were carried out to evaluate the gel time for different amounts of crosslinking agent. The microstructure of the aerogels was investigated by scanning electron microscopy (SEM, TM4000Plus II)) analyses. XRD analyses were carried out using a X’Pert Pro X-ray diffractometer (Malvern Panalytical), equipped with a PIXCel 1D detector. To get reliable results, the aerogel samples were prepared in the form of films (a representative picture of the obtained sample is given in the Supplementary Materials, Section S1). The 2θ scan range was 5–60° with a step size of 0.01°. The dye removal ability of the aerogels was investigated by batch experiments carried out in a thermostated shaker (SKI 4, Argo Lab) at 150 rpm and 25 °C. Mono-component solutions at initial concentration ranging from 50 to 750 mg L^−1^ were obtained by diluting stock solutions (1000 mg L^−1^), which were obtained by dissolving the dye powder in bi-distilled water under magnetic stirring for 5 h without any pH adjustment. The adsorption capacity at equilibrium, *q_e_*, was estimated as:

(1) $$q_{e}=\left(C_{0}-C_{e}\right)\frac{V}{m}$$

where *C_0_* and *C_e_* represent the dye concentration at the beginning and at the end of the adsorption experiments, respectively, *V* is the volume of the dye solution (10 mL), and *m* is the adsorbent mass (10 mg). Preliminary tests were carried out to assess the time needed to reach equilibrium conditions, which was about 3 h. The *C_e_* values were measured by analyzing the solutions with a UV-vis spectrophotometer at the characteristic maximum absorbance wavelength (Supplementary Materials, Section S2): 611 nm for the anionic dye indigo carmine (IC) and 527 nm for the cationic dye rhodamine 6G (RH). The dye concentration was evaluated for each test before and after the addition of the adsorbent sample. If necessary, dye solutions were preventively diluted before UV-vis spectroscopy analysis, in order to apply the Lambert–Beer law.

## 3. Results and Discussion

### 3.1. Optimization of the Preparation Process

The chemical crosslinking of chitosan represents a crucial step in the preparation process. Indeed, a high crosslinking degree is essential to ensure mechanical and chemical stability of the aerogels, especially when they are soaked in water for adsorption tests. On the other hand, it implies that part of the functional groups of chitosan (mainly, hydroxyl and amine groups) that are involved in the crosslinking reaction cannot be available as active sites for adsorption, thus lowering the overall performances of the resulting material. In addition, the crosslinking strategy has recently been found to play a key role in determining the adsorption properties of CS-based aerogels [25]. In particular, the dye removal ability can be improved if the crosslinking step is carried out after the freeze-drying process. According to all these observations, the amount of crosslinker, namely glutaraldehyde (GA), to be added to the CS/LTA dispersion was selected to meet the following trade-off requirements:

(i) it has to be present at a low enough concentration so as to avoid the gelation of the dispersion before it is completely frozen in the refrigerator;
(ii) it has to be present at a high enough concentration so as to allow the crosslinking reactions to occur in freeze-dried samples subjected to a thermal treatment (see Section 3.1 for details on the aerogel preparation protocol).

**Figure 2:**
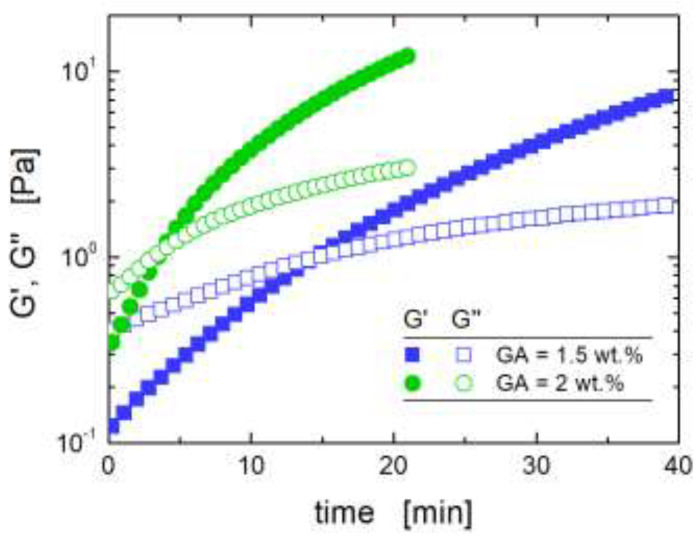
Time dependence of the elastic (full symbols) and loss (empty symbols) modulus at a frequency of 1 rad s^−1^ for aqueous CS/LTA (weight ratio equal to 2:1) dispersions crosslinked at 25 °C with 2 (circles) and 1.5 (squares) wt.% of GA.

To verify the fulfillment of requirement (i), rheological analyses were carried out, in order to define the time window available before gelation in the CS/LTA dispersions. A representative output of the measurements is shown in Figure 2.

In a typical test, both the elastic, G’, and loss, G’’, moduli increase over time, the growth of G′ being more pronounced because of the occurrence of the crosslinking reactions. An indicative estimate of the gel time is represented by the time where G′ reaches and eventually surpasses G′′ [26]. Crosslinking kinetics is obviously faster when CS concentration is higher, hence, CS/LTA dispersion with the highest investigated weight ratio (i.e., CS:LTA = 2:1) was used, as a conservative reference to set the crosslinker amount. According to the results of the viscoelastic analyses, the optimal GA amount for the aerogel preparation was set to 1.5 wt.%, for which a gel time of about 15 min was found by viscoelastic measurements. Indeed, a time window of about 7 min was found to be enough to freeze 1 cm^3^ (approximate sample size) of CS/LTA aqueous dispersion at −25 °C. The CS/LTA aerogels were finally obtained by subjecting the freeze-dried dispersions to a thermal treatment (see Section 3.1 for details).

### 3.2. Structure of the Aerogels

**Figure 3:**
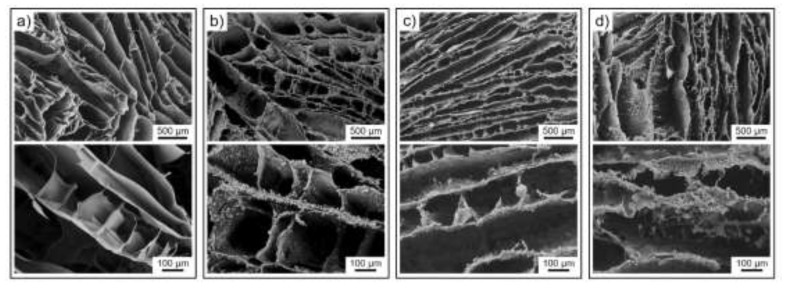
SEM micrographs showing the cross-section of (**a**) pristine CS and (**b**–**d**) hybrid CS/LTA aerogels at different weight ratios: (**b**) 2:1, (**c**) 1:1, and (**d**) 1:2.

The microstructure of the hybrid aerogels at different CS/LTA weight ratios, obtained after thermal treatment and crosslinking, is shown in the SEM micrographs reported in Figure 3.

**Figure 4:**
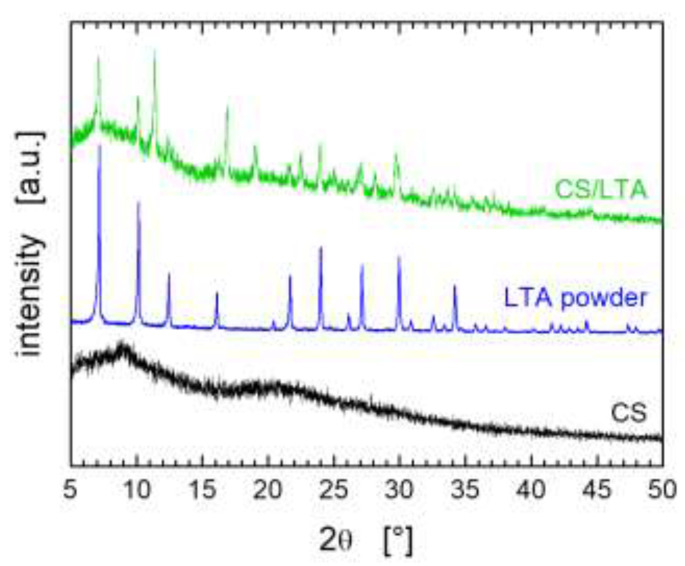
XRD patterns of CS, LTA powder, and hybrid CS/LTA sample at weight ratio equal to 1:1. The XRD patterns have been vertically shifted for the sake of clarity.

The samples share the same overall porous structure with hundred-micron wide and mostly disordered lamellar channels, which results from the adopted freezing conditions [27,28]. The thickness of the lamellae increases from around 10 μm to around 30 μm as the content of LTA is increased. The thickness of the lamellae has relevant implications since it determines the kinetics of dye adsorption which is expected to occur not only on the surface of the channels but also within each lamella. The characteristic size of the porous channels is not significantly affected by LTA particles. Their presence instead can be clearly appreciated in the SEM micrographs of the hybrid aerogels at higher magnification (Figure 3b–d, bottom row), in which micrometer-sized particles decorate the wall of the CS framework. By increasing the LTA content in the aerogel formulation, the pore walls change from partially covered by LTA particles (CS/LTA ratios of 2:1, Figure 3b) to fully armored by an inorganic shield (CS/LTA ratios of 1:1 and 1:2, Figure 3c,d). The presence of LTA zeolite in the hybrid aerogels and the preservation of its structure at the end of the preparation process was also verified by X-ray diffraction (XRD) analysis. The XRD pattern of a representative CS/LTA sample is shown in Figure 4, together with the diffractograms of pristine LTA powder and CS aerogel to be used as references (additional XRD spectra are provided in the Supplementary Materials, Section S1).

Figure 4 clearly shows that the crystalline nature of the zeolite is preserved in the hybrid aerogel, as evidenced by the presence of the characteristic main peaks of LTA powder also in the CS/LTA diffractogram. It can be also noted a sharp peak at around 11.4° indicating the presence of sodium acetate, which results from the pH adjustment to ~5 with few drops of NaOH solution before adding the zeolite powder to the CS solution (see the experimental section for details on the procedure). The complete peak assignment for the CS/LTA zeolite hybrid aerogel is provided in the Supplementary Materials, Section S1.

### 3.3. Adsorption Properties

**Figure 5:**
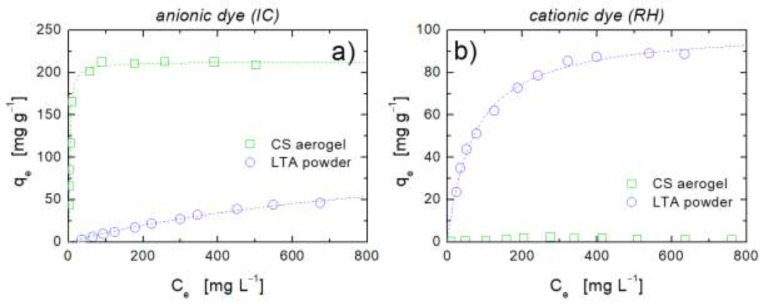
Adsorption isotherms at 25 °C of (**a**) anionic dye IC and (**b**) cationic dye RH for pristine CS aerogels (squares) and LTA powder (circles). Dashed lines represent best fitting with the Langmuir model (Equation (1)).

In the analysis of adsorption properties, we first consider the adsorption isotherms of the anionic dye, IC, and cationic dye, RH, for pristine CS aerogels and LTA powder, that are reported in Figure 5a,b.

As expected, the protonated amino groups of chitosan bring about a remarkable adsorption of the anionic pollutant thanks to the electrostatic interactions setting up with the ${-\mathrm{SO}}_{3}^{-}$ groups on the IC dye molecule (Figure 5a), which is in line with other studies on the same polymer-dye pair [15,29,30]. At the same time, the strong electrostatic repulsion between the positively charged chitosan chains and the cationic RH dye (${-\mathrm{NH}}^{+}$ group) results in a barely detectable adsorption isotherm (Figure 5b). On the other hand, zeolite powders have been widely employed mainly for the removal of cationic dyes [31,32,33], and only a few studies reported a rather low adsorption capacity towards anionic species [34,35]. This is in qualitative agreement with the results showed in Figure 5, which highlight that for the LTA powder, the adsorbate–adsorbent interactions are in fact larger in the case of the cationic pollutant. For both IC and RH, the molecular size of the dyes [15,36] largely exceeds the pore size of LTA zeolite containing sodium ions, which is about 0.4 nm [37]. This indicates that ion-exchange processes can be ruled out, and that the LTA powder mainly interacts with dye molecules via electrostatic forces. In particular, the negatively charged surface of LTA mostly interacts with the positively charged groups of cationic dyes. Hydrogen bonding between nitrogen atoms of the dye and silanol group of zeolite is mainly responsible for the adsorption of the anionic dye [38].

All the experimental data reported in Figure 5 are adequately interpreted by the Langmuir equation [39]:

(2) $$q_{e}=\frac{q_{e}^{max}bC_{e}}{1+bC_{e}}$$

where $q_{e}^{max}$ (mg g^−1^) and b (L mg^−1^) represent the maximum adsorption capacity and the so-termed “energy of adsorption”, respectively. The values of these parameters resulting from data fitting are reported in Table 1. Notably, the adsorption of IC in CS is much more favorable than the adsorption of RH in LTA (see higher values of $q_{e}^{max}$ and b for the former case). The fitting parameters could not be properly evaluated in the case of adsorption of cationic dye by CS aerogel because of the low values of dye uptake, which resulted in a poorly reliable fitting (see Figure 5b).

We will use the isotherms in Figure 5 for comparison with the results obtained with hybrid aerogels. The simplest approach to interpret adsorption isotherms of adsorbates in CS/LTA aerogels is by assuming an additive behavior of adsorption, expressed by the following equation:

(3) $$q_{e}=\frac{q_{e,CS}^{max}b_{CS}C_{e}}{1+b_{CS}C_{e}}f_{CS}+\frac{q_{e,LTA}^{max}b_{LTA}C_{e}}{1+b_{LTA}C_{e}}\left(1-f_{CS}\right)$$

where $q_{e,CS}^{max}$ and $q_{e,LTA}^{max}$ are the adsorption capacities of the adsorbate in CS and LTA, $b_{CS}$ and $b_{LTA}$ are the energies of adsorption of the adsorbate in CS and LTA, while $f_{CS}$ is the mass fraction of chitosan in the CS/LTA hybrid aerogel.

**Figure 6:**
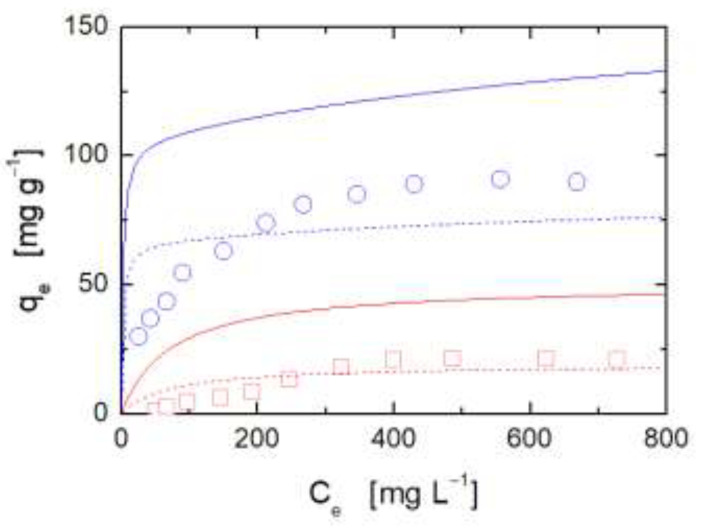
Adsorption isotherms at 25 °C of anionic dye IC (blue circles) and cationic dye RH (red squares) for hybrid CS/LTA aerogel at weight ratio equal to 1:1. Solid lines correspond to Equation (3), and dashed lines represent the best fitting with Equation (4). In both cases, Langmuir model parameters of pristine CS and LTA reported in Table 1 have been considered.

Figure 6 shows the experimental adsorption isotherms of both investigated dyes in the hybrid CS/LTA aerogel at a weight ratio equal to 1:1 along with prediction obtained by using Equation (3) (solid lines).

Evidently, the additive model largely overestimates the adsorbed amounts. This finding points to the occurrence of cross-interactions between chitosan and zeolite that reduce the number of sites available for adsorption that are present on chitosan macromolecules and zeolite particles. This results in a reduction of adsorption capacity in the case of hybrid aerogel with respect to that expected from the combination of the adsorption capacity of the pristine constituents. In fact, as reported in the literature [40,41,42], cross-interactions could establish between amine-nitrogen electrons of chitosan and surface aluminum ions of zeolite, and, in addition, concurrent hydrogen bonding could establish between amino and –OH hydrogens on chitosan and the –OH surface groups of zeolites. Moreover, chitosan could result in steric constriction that would hinder the access to the zeolite surface. These features of the hybrid aerogel are expected to reduce the interactions of amine groups of chitosan with anionic dye, and to reduce the adsorption capacity of both kinds of dyes in the zeolite particles.

To account for the impact of these interactional and steric effects on the adsorption isotherms of hybrids, a mixing rule for CS and LTA adsorption isotherms, slightly more complex than Equation (3), has been also considered:

(4) $$q_{e}=\frac{(q_{e,CS}^{max}-x)b_{CS}C_{e}}{1+b_{CS}C_{e}}f_{CS}+\frac{(q_{e,LTA}^{max}-x)b_{LTA}C_{e}}{1+b_{LTA}C_{e}}\left(1-f_{CS}\right)$$

This expression is based on the assumption that the cross-interactions between CS and LTA result in a symmetric decrease of the adsorption capacity of both components (accounted for by the parameter ‘*x*’), while the interaction energy parameters are not affected. Obviously, more complex mixing rules could be used, introducing additional parameters into the model, possibly accounting also for energetic effects. However, we think that available data do not justify the introduction of additional meaningful parameters to Equation (4). The results of fitting of adsorption isotherms of both kinds of dyes for CS/LTA aerogel at weight ratio equal to 1:1 are reported in Figure 6 (dashed lines). A better interpretation of data has been reached as compared to the predictions provided by Equation (3), with an estimated value of ‘*x*’ equal to 6.25 × 10^1^ mg g^−1^. Equation (4) still fails to provide a satisfactory interpretation of experimental data, and in particular, does not capture the right curvature of the isotherms. Nevertheless, this approach qualitatively confirms the reduction in adsorption capacity promoted by the physical–chemical interaction between the two components of the hybrid aerogels.

**Figure 7:**
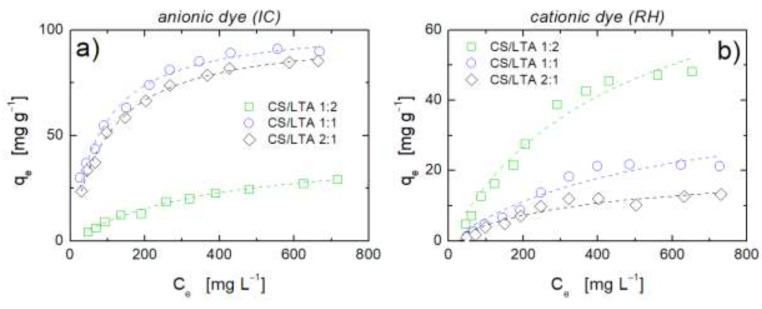
Adsorption isotherms at 25 °C of (**a**) anionic dye IC and (**b**) cationic dye RH for hybrid aerogels at different CS/LTA weight ratios: 1:2 (squares), 1:1 (circles), and 2:1 (diamonds). Dashed lines represent best fitting with the Langmuir model (Equation (2)).

In the following, we report the overall results in terms of adsorption isotherms obtained with the three investigated hybrids (CS/LTA ratio 1:2, 1:1 and 2:1) for both types of dyes. Experimental results are reported in Figure 7 along with data fitting performed by using the Langmuir equation (Equation (2)) for each of the investigated samples. A good fitting has been obtained and the model parameters are reported in Table 2. The maximum adsorption capacity calculated by considering an additive behavior of adsorption, *q_e_^calc^*, from data in Table 1 is also reported as reference value.

The analysis of the values of the Langmuir’s parameters reported in Table 2 allows some considerations to be formulated regarding the adsorption features of hybrid aerogels. Examining first the adsorption of the anionic dye, IC, it is evident how $q_{e}^{\max}$ is considerably lower than the sum of the adsorption capacity of the two components of the hybrids, confirming the reciprocal frustration of adsorption capacity. Notably, adsorption energy is remarkably low as compared to pure CS, even at the higher percentage of CS in the hybrid, thus indicating that there is an effect of the CS-LTA combination on the strength of the interaction of active sites on chitosan with the IC, likely due to the partial shielding of these groups. This conclusion provides further indication of the inadequacy of Equation (4) in interpreting adsorption isotherms. Quite interestingly, both the adsorption capacity and the adsorption energy seem to reach a maximum or a plateau value while increasing the CS content.

The analysis of the parameter values determined for the case of adsorption of the cationic dye, RH, in hybrid aerogels also provides some food for thought. In the case of RH, only LTA is expected to contribute to the adsorption capacity of the aerogel hybrids. Notably, the adsorption capacity of the zeolite is more affected the higher the chitosan content. In fact, for a CS/LTA ratio of 1:2, the adsorption capacity, after accounting for the experimental error, is close to what one would expect, on the basis of composition, by the sole LTA. The CS/LTA aerogel with 1:2 composition, indeed, resulted in a maximum RH adsorption of ~90 mg g^−1^, which is not far from the maximum uptake of the pure LTA powder (~100 mg g^−1^). These findings highlight how the hindering effect of CS on LTA is effective only above a threshold composition of the hybrid. In this case also, evidence of a shielding effect is present, in view of the significantly reduced value of interaction energy as compared to the case of pure LTA.

Further insights on how molecular interactions between chitosan and zeolite affect the availability of active sites for dye molecules adsorption may also be provided by numerical simulations. For instance, recent molecular dynamics approaches focus on the dynamics of polymers at the interface with chemical heterogeneous solids [43,44,45,46]. These models can be properly modified to study how chemical heterogeneity affects the adsorption processes involved in dye removal.

A comparison with adsorption data taken from literature for other CS/zeolite composite systems is provided in Table 3. Overall, our hybrid aerogels exhibit adsorption capacity values that are in line with the average of comparable materials. Nonetheless, it is worth noting that only few other successful examples exist in which the complementary adsorption behavior of chitosan and zeolite is profitably combined in a single adsorbent, which possesses a satisfactorily removal ability towards both anionic and cationic dyes. Among others, it is worth mentioning the results achieved by Khanday et al. for composite beads made of chitosan and activated oil palm ash zeolite, which exhibit a *q_e_^max^* higher than 100 mg g^−1^ for two oppositely charged dyes [3]. In other cases, zwitterionic adsorbents have been developed, but their effectiveness towards anionic or cationic molecules depends on the specific temperature and/or pH conditions at which they are employed [47].

## 4. Conclusions

Hybrid aerogels made of chitosan and Linde Type A zeolite particles were obtained by freeze casting of an aqueous CS/LTA dispersion. Crosslinking with GA was performed by subjecting the aerogels to thermal treatment. The effect of the CS/LTA ratio on the adsorption performances towards anionic (indigo carmine) and cationic (rhodamine 6G) dyes was investigated through adsorption isotherms by batch tests. Overall, the produced hybrid aerogels ensure satisfactory performances for the adsorption of both anionic and cationic dyes. It should be noted that the dye uptake capacity was assessed in nearly neutral conditions, without the need of pH adjustments to improve adsorption in acidic/alkaline conditions. Depending on the aerogel composition, a tradeoff of the adsorption performance is obtained. At higher CS content, the removal of IC is higher, while at higher LTA fraction, the RH uptake is higher: as a result, the best compromise is obtained with the aerogel at a CS:LTA weight ratio equal to 1:1. However, its efficiency appears to be lower than those resulting from the additivity rule of the separate components. This result indicates that molecular interactions between chitosan and zeolite play an important role, leading, for example, to a local reduction of active sites for dye molecules adsorption. The chemical heterogeneity arising from close patches of different active sites can also be relevant, likely resulting in screened interactions between adsorbent and dyes. In addition, steric effects and their implications on the local morphology may also be at play. In order to elucidate these issues, it would be interesting to deepen the study on the density and availability of active site before and after mixing the two aerogel constituents.

## Figures and Tables

**Table 1:** Langmuir model best fitting parameters for adsorption isotherms for CS aerogels and LTA powder.

Adsorbate	Adsorbent	*q_e_^max^* [mg g^−1^]	*b* [L mg^−1^]	*R* ^2^
IC	CS aerogel	212.8 ± 3.4	0.4239 ± 0.0398	0.986
IC	LTA powder	124.5 ± 15.1	0.0010 ± 0.0001	0.992
RH	LTA powder	101.1 ± 1.5	0.0141 ± 0.0008	0.994

**Table 2:** Langmuir model best fitting parameters for adsorption isotherms for hybrid CS/LTA aerogels.

Adsorbate	Adsorbent	*q_e_^calc^* [mg g^−1^]	*q_e_^max^* [mg g^−1^]	*b* [L mg^−1^]	*R* ^2^
Anionic dye (IC)	CS/LTA 1:2	152.4	44.7 ± 2.0	0.0026 ± 0.0002	0.992
	CS/LTA 1:1	168.7	103.1 ± 2.3	0.0125 ± 0. 0010	0.985
	CS/LTA 2:1	181.5	98.9 ± 1.4	0.0103 ± 0.0005	0.995
Cationic dye (RH)	CS/LTA 1:2	67.4	89.3 ± 11.8	0.0021 ± 0.0005	0.966
	CS/LTA 1:1	51.6	43.4 ± 10.3	0.0017 ± 0.0005	0.917
	CS/LTA 2:1	34.7	20.6 ± 3.6	0.0027 ± 0.0010	0.899

**Table 3:** Comparison of the dye adsorption capacity of CS/LTA aerogels with data reported in the literature.

Adsorbent	Anionic Dye(s)	*q_e_^max^* [mg g^−1^]	Cationic Dye(s)	*q_e_^max^* [mg g^−1^]	Ref.
Alginate beads with magnetic Chitosan-Zeolite	-	-	Methylene blue	6	[48]
Chitosan/zeolite A film	B. Orange 16	305	-	-	[1]
Chitosan/polyvinyl alcohol/zeolite membrane	Methyl orange	153	-	-	[49]
Chitosan/zeolite composite	Reactive Red 120 and 196	19.1, 35.6	-	-	[50]
Activated oil palm ash zeolite/chitosan beads	Acid blue 29	213	Methylene blue	152	[3]
CS/LTA 1:1 aerogel	Indigo carmine	103	Rhodamine 6G	43.4	This study

## Data Availability

The data presented in this study and not reported in tables are available on request from the corresponding author.

## Supplementary Materials

Additional data and information on XRD analyses UV-vis spectroscopy are given as Supplementary Materials and can be found at https://www.mdpi.com/article/10.3390/ijms22115535/s1.
