# Supplementary material for: Tailoring Chitosan/LTA Zeolite Hybrid Aerogels for Anionic and Cationic Dye Adsorption
Source: Int J Mol Sci. 2021 May 24;22(11):5535. doi: 10.3390/ijms22115535 (PMC8197200; doi:10.3390/ijms22115535)
Supplement: Supplementary file 1 [file ijms-22-05535-s001.zip › ijms-1219011-supplementary.pdf]

Tailoring Chitosan/LTA Zeolite Hybrid Aerogels for Anionic and Cationic Dye Adsorption

Martina Salzano de Luna [1], Francesco Greco [1], Raffaele Pastore [1], Giuseppe Mensitieri [1], Giovanni Filippone [1], Paolo Aprea [1], Domenico Mallamace [2], Francesco Mallamace [3] and Sow-Hsin Chen [3]

[1] Department of Chemical, Materials and Production Engineering, University of Naples Federico II, Piazzale Tecchio 80, 80125 Napoli, Italy.

[2] Departments of ChiBioFarAm and MIFT- Section of Industrial Chemistry, University of Messina, CASPE-INSTM, V.le F. Stagno d'Alcontres 31, 98166 Messina, Italy.

[3] Department of Nuclear Science and Engineering, Massachusetts Institute of Technology, Cambridge, MA 02139, USA.

[*] Correspondence: martina.salzanodeluna@unina.it; Tel.: +39-081-768-2407 (M.S.d.L.)

S1. XRD analyses

An image showing a representative sample used for XRD analyses is given in Figure S1. In particular, the samples were obtained by casting the CS solution or CS/zeolite dispersion on a microscope glass slide, which was then freeze-dried to get aerogel films. In this regard, we also note that the presence of voids (i.e. macroporosity) and the inherent inhomogeneity of the samples is responsible for the relatively low peak-to-background ratio in the CS and CS/LTA patterns

reported in Figure 3 of the main text. The XRD spectra of the CS/LTA aerogels at different composition are given in Figure S2.

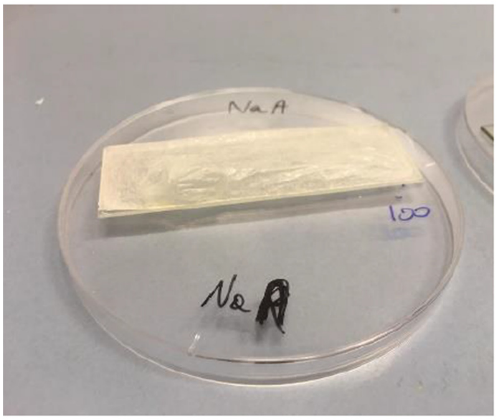

Figure S1. Example of film of chitosan/LTA zeolite hybrid aerogel deposited on a glass slide.

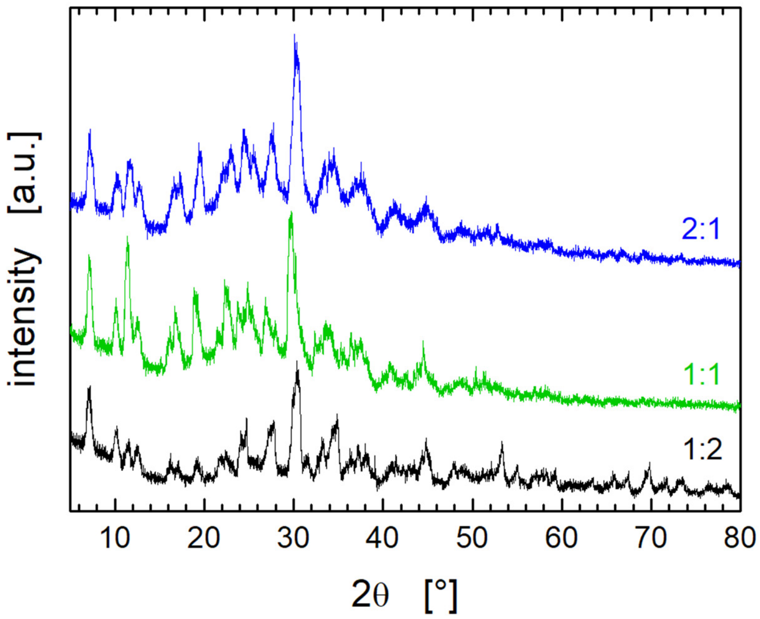

Figure S2. XRD patterns of CS/LTA sample at different CS:LTA weight ratio. The XRD patterns have been vertically shifted for the sake of clarity.

Peak assignation for the XRD pattern of hybrid aerogels was performed by using PANalytical HighScore and ICCD PDF-2 database by looking for zeolite LTA. The presence of sodium acetate hydrate, resulting from of acetic acid and sodium hydroxide in solution, was hence deduced. A representative output is given in Figure S3.

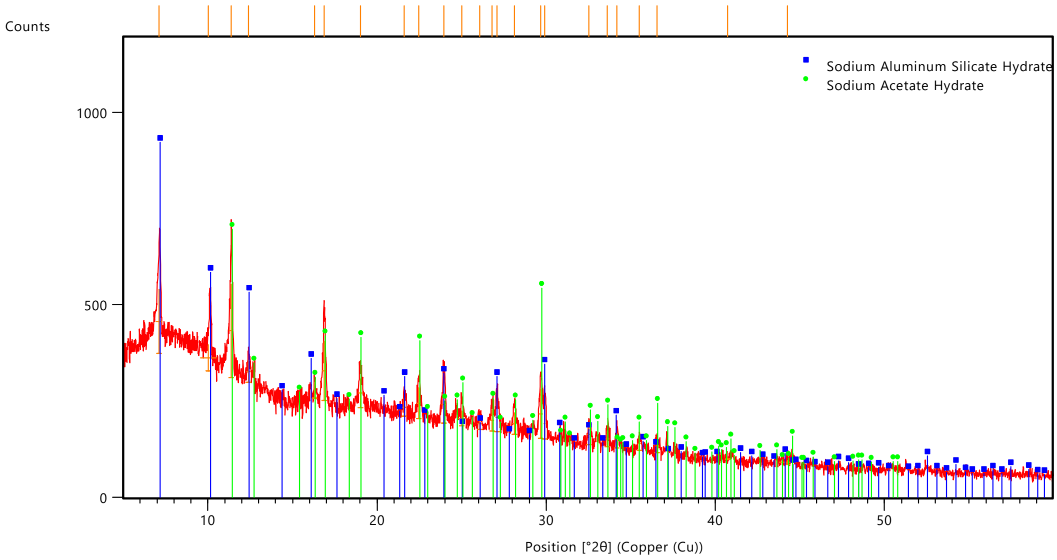

Figure S3. Peak assignation for the CS/LTA 1:1 hybrid aerogel. The two identified crystalline phases correspond to LTA zeolite (blue squares) and sodium acetate hydrate (green triangles).

S2. UV-vis spectroscopy

The UV-vis spectra of IC and RH aqueous solutions (~$2 \cdot 10^{-3}$ mg ml^{-1}) are shown in Figure S4. The absorbance peak was used as reference for application of the Lambert-Beer law.

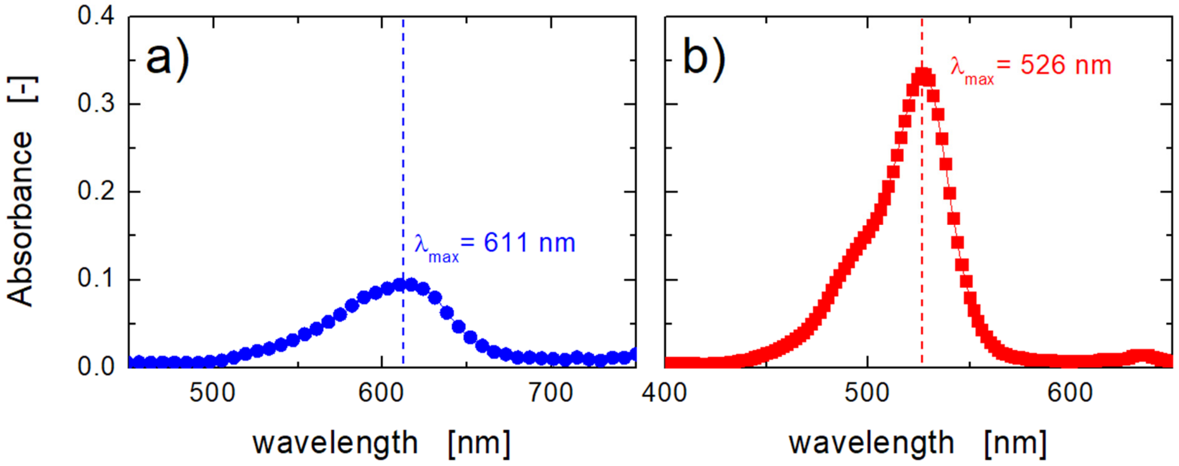

Figure S4. UV-vis spectrum of a) IC and b) RH aqueous solutions.
